# Depressive Symptoms and Cognitive Decline Among Chinese Rural Elderly Individuals: A Longitudinal Study With 2-Year Follow-Up

**DOI:** 10.3389/fpubh.2022.939150

**Published:** 2022-07-13

**Authors:** Shuai Zhou, Qiong Wang, Jingya Zhang, Qing Wang, Fangfang Hou, Xiao Han, Shilian Hu, Guodong Shen, Yan Zhang

**Affiliations:** ^1^Department of Health Service Management, School of Health Service Management, Anhui Medical University, Hefei, China; ^2^Department of Geriatrics, The First Affiliated Hospital of University of Science and Technology of China, Gerontology Institute of Anhui, Division of Life Sciences and Medicine, University of Science and Technology of China, Hefei, China; ^3^Anhui Key Laboratory of Tumor Immunotherapy and Nutrition Therapy, Hefei, China

**Keywords:** depression, mild cognitive impairment, elderly, rural area, Alzheimer's disease

## Abstract

**Background:**

Depressive symptoms and mild cognitive impairment (MCI) are highly prevalent in rural China. The study aimed to investigate the longitudinal associations between changes in depressive symptoms and cognitive decline and MCI incidence among Chinese rural elderly individuals.

**Methods:**

A 2-year follow-up study was conducted among 1,477 participants from the Anhui Healthy Longevity Survey (AHLS). Depressive symptoms were assessed by the 9-item Patient Health Questionnaire (PHQ-9), and cognitive status was evaluated by the Mini Mental State Examination (MMSE). Multivariable linear regression and logistic regression were employed.

**Results:**

Every 1-unit PHQ-9 score increase was significantly associated with more cognitive decline (β = 0.157, 95% CI: 0.092, 0.221, *p* < 0.001) and a higher risk of MCI incidence (OR = 1.063, 95% CI: 1.025, 1.103, *p* = 0.001). The participants who experienced worsening of depression symptoms had a larger decline in the 2-year MMSE score (β = 0.650, 95% CI: 0.039, 1.261, *p* = 0.037) and elevated risks of incident MCI (OR = 1.573, 95% CI: 1.113, 2.223, *p* = 0.010).

**Limitations:**

Screening tools rather than standard diagnostic procedures were used in the study. Moreover, the long-term associations still need further exploration since the follow-up time was short.

**Conclusions:**

Increased depressive symptoms were associated with more cognitive decline and higher risks of incident MCI among Chinese rural residents.

## Introduction

Alzheimer's disease (AD) is a significant and common public health threat worldwide, not only creating enormous physical, emotional and economical stress for individuals and their families but also imposing a very large disease burden on societies. In China, the number of people living with AD has been projected to be 300 million, with a disease burden as high as 2000 billion RMB in 2050 (1). Due to the slow progress toward curing AD, preventive strategies through elucidating and modifying its risk factors might be the key response initiatives to combat AD (2). Mild cognitive impairment (MCI) has been regarded as a predementia stage of great public health importance (3, 4), because individuals with MCI have the potential to regain normal cognitive function if early and effective interventions can be taken (5).

Depression and cognitive decline often present together (6–8), indicating the possible close relationship between them, although the exact mechanism seems to be complicated and needs further exploration. Many studies have suggested that depressive symptoms increase the risk of cognitive impairment through pathways of immune dysregulation, which may simultaneously drive other comorbid medical conditions (9, 10). However, some researchers hold the view that depression is one of the prodromes of cognitive decline because people with depression show a negative bias in information processing that affects their attention, memory, and response to feedback (11). Additional evidence has revealed that depressive symptoms play a major role in the progression of MCI to AD (12, 13); thus, depression has been regarded as a predictor of progressive cognitive decline (14, 15).

Despite relatively numerous studies on the relationship between depression and cognitive decline, a few gaps exist in the literature. First, although depression is very common among elderly individuals (16), available studies are often limited to middle-aged people (17), clinical patients (18), and urban areas (19, 20). In addition, longitudinal studies, which are essential to investigate the causal effect of depression on the onset of cognitive decline, are still insufficient (21, 22). Although several longitudinal studies have implied a relationship between depression and cognitive decline (23, 24), evidence from less developed areas, e.g., rural China, is still very rare.

Therefore, in the current study, we aimed to explore the relationship between depression and cognitive decline and the incidence of MCI among a rural Chinese elderly sample through a longitudinal study with 2-year follow-up, particularly to determine whether baseline depression and depression status change were associated with cognitive decline and MCI incidence. The findings of the current study might contribute to preventing MCI and slowing the development of AD among older adults in rural areas.

## Methods

### Study Population

The present study was based on data collected from the Anhui Healthy Longevity Survey (AHLS), the details of which have been reported elsewhere (25). Briefly, 6,211 participants who were aged 60 or higher were initially enrolled from four cities (Chuzhou, Lu'an, Xuancheng, and Fuyang) in July 2019. After approximately two years, the follow-up visit (wave 1) was conducted the participants from rural areas of the three cities (Chuzhou, Lu'an and Xuancheng). Among the 2,308 rural participants who were investigated at baseline, 1,566 participants completed the follow-up in 2021, for a response rate of 67.9% (742 participants were lost to follow-up, including 88 cases of death, 23 cases of loss of communication ability, 201 cases of migration and 430 cases of refusal to participate).

In the current study, we further excluded the participants if they (1) were not able to finish the cognitive function assessment or depressive symptoms assessment (*n* = 16) or (2) had any missing covariate data (*n* = 73). Finally, 1,477 participants were included in the analysis, and none of them reported a previous history of depression or cognitive impairment. For the analysis of the relationship between depression and 2-year MCI incidence, the participants with pre-existing MCI at baseline (*n* = 983) were excluded. All the procedures complied with the ethical standards of the Anhui Medical University committee (No. 2020H011).

### Assessment of Depressive Symptoms

The Patient Health Questionnaire (PHQ-9) (26) was used to measure the depression symptoms of the participants at baseline and at follow-up. The instrument assessed the severity of depression by asking about the frequency of occurrence of the major symptoms of depression within the last two weeks. The total score of the PHQ-9 is 30, with higher scores indicating more severe depression symptoms. The Cronbach's α was 0.89 in the pilot study, showing the appropriate internal consistency of the PHQ-9 scale. For the current analysis, total PHQ-9 scores were categorized as “not depressed” (0–4) and “depressed” (5–30).

The depression score change was calculated by the formula

***i*** = *P* − *Q* *where, P* = PHQ-9 score at follow up; *Q* = PHQ-9 score at baseline.

A greater value of ***i*** indicates an increase in PHQ-9 scores at follow-up, i.e., more elevated depressive symptoms. Worsening depressive symptoms were classified into “None” (***i*** was ≤ 0) and “Yes” (***i*** was higher than 0).

### Assessment of Cognitive Function

The cognitive status of the participants was evaluated by the Mini Mental State Examination (MMSE) (27) at baseline and at follow-up. The MMSE was the most widely used tool for cognition assessment, with good accuracy in the detection of dementia (sensitivity and specificity were 87 and 89%, respectively) (28) as well as in MCI (sensitivity and specificity were 79.8 and 81.3% respectively) (29). A previous study concluded good applicability of MMSE as a cognitive function screen instrument in community settings of China (30). The total score of the MMSE scale is 30 points, with higher scores indicating better cognitive function. The Cronbach's α was 0.69 in the pilot study, showing the acceptable internal consistency of the MMSE. In the current study, the education-based criterion for MCI was adopted due to the potential impact of educational level (31). Specifically, MCI was determined if the participants were illiterate and had MMSE scores <18, if they had 1–6 years of education and had MMSE scores lower than 21, or if they had 6 or more years of education with MMSE scores lower than 25 (32).

Two-year cognitive change and MCI incidence were also identified according to the measurement of MMSE scores at baseline and at follow-up. The changes in MMSE scores were calculated by the formula

*j* = *M E* *where, M* = MMSE score at baseline; *E* = MMSE score at follow-up.

A greater value of *j* indicates a larger decrease in the MMSE score after 2 years of follow-up, i.e., a severe decline in cognitive performance.

The participants who were free of MCI at baseline and classified as having MCI at follow-up were judged as having incident MCI. The participants were divided into two groups (yes, no) according to whether they developed MCI within the 2-year follow-up.

### Assessment of Covariates

Baseline data of sociodemographic characteristics (city, sex, age, education, marital status, annual income, and living alone), behavioral factors [body mass index (BMI), drinking, smoking, sedentary hours, self-rated sleep quality], and chronic conditions (diabetes and hypertension) were treated as covariates. Age was self-reported by the participants. Education was divided into three groups according to the participant's years of formal education (low: 0, medium: 1–6 years and high: more than 6 years). Marital status was classified into married and others (including divorce, widowed and never married). Annual income was classified into two groups (lower than 6,500 RMB and 6,500 RMB or higher). Living alone was assessed by asking the participants “Do you live alone?” The options were “yes” and “no.” BMI was calculated by measuring height and weight by the formula *weight (kg)/height (m)*^2^ and introduced as a continuous variable. Drinking status was dichotomized as never, former, and current. Smoking was classified into two groups (yes and no) according to whether they smoked. Self-rated sleep quality was assessed by asking the participants “How do you evaluate your sleep quality in the last month?” The options were “very good,” “good,” and “not good.” Continuous daily sitting time was self-reported and used as an indicator reflecting the physical activity status of the participants. Two chronic conditions (hypertension and diabetes) were divided into two groups (yes and no) according to whether the participants had been diagnosed with hypertension/diabetes at a hospital at the county level or above.

### Statistical Analyses

Multivariable linear regression and logistic regression were employed to assess the associations between depressive symptoms and cognition by including 2-year cognitive decline and MCI incidence as dependent variables, respectively. The baseline PHQ-9 score and changes in PHQ-9 scores (***i***) were treated as independent variables and introduced into the regression models together with all the covariates. The regression models used simultaneous entry of variables (enter method). Since many studies have indicated that sex and age might be related to cognitive decline and depression (33–35), sex- and age-stratified analyses were also performed. In addition, accumulating evidence suggested that having more years of education is a protective factor for cognitive impairment (36, 37); thus, additional analyses stratified by education levels were also conducted to examine the associations in different subgroups.

Stata version 15.1 software (Stata Corp, College Station, TX) was used for all analyses. All the tests were two-sided, and the significance level was set at *p* < 0.05.

## Results

Table 1 presents the basic characteristics of the study sample (*n* = 1,477). The mean age of the participants was 70.36 years, and 51.9% of the participants were women. More than half of the participants (56.8%) enrolled in the current study were illiterate. The mean BMI of the participants was 23.79 kg/m^2^. A total of 14.4% of the participants reported having diabetes, and more than half of the participants (52.3%) reported having high blood pressure.

**Table 1:** Basic characteristics of the sample.

	***N***	**Mean/** **percentage**
**City**
Lu'an (West)	488	33.0
Chuzhou (East)	568	38.5
Xuancheng (South)	421	28.5
**Sex**
Male	711	48.1
Female	766	51.9
**Age**	1,477	70.36
**Education**
Low (0 year of education)	839	56.8
Medium (1~6 years of education)	480	32.5
High (more than 6 years of education)	158	10.7
**Marital status**
Married	1,097	74.3
Others (divorce/widowed/Never married)	380	25.7
**Annual income**
<6,500	1,168	79.1
≥6,500	309	20.9
**BMI**	1,477	23.79
**Drinking status**
Never	785	53.2
Former	71	4.8
Current	621	42.0
**Current smoker**
Yes	371	25.1
No	1,106	74.9
**Physical activity**
Daily sitting time (hours)	1,477	4.12
**Self-rated sleeping quality**
Very good	256	17.3
Good	843	57.1
Not good	378	25.6
**Diabetes**
Yes	212	14.4
No	1,265	85.6
**Hypertension**
Yes	773	52.3
No	704	47.7
**Living alone**
Yes	259	17.5
No	1,218	82.5

The associations between depression status at baseline and at follow-up and cognitive function are shown in Table 2. Depression status at baseline was not associated with two-year cognitive decline or MCI incidence. For depression status at follow-up, every 1-unit PHQ-9 score increase was significantly associated with a larger 2-year cognitive decline (β = 0.157, 95% CI: 0.092, 0.221, *p* < 0.001) and a higher risk of MCI incidence (OR = 1.063, 95% CI: 1.025, 1.103, *p* = 0.001). The participants who experienced worsening depressive symptoms had a larger decline in the 2-year MMSE score (β = 0.650, 95% CI: 0.039, 1.261, *p* = 0.037) and elevated risks of incident MCI (OR = 1.573, 95% CI: 1.113, 2.223, *p* = 0.010).

**Table 2:** The associations between depression and cognitive function: Coefficients and 95% CIs for 2-year cognitive decline and Odds Ratios and 95% CIs for 2-year MCI incidence.

	**2-year cognitive decline (** ***j*** **)** ^a^			**2-year MCI incidence** ^b^		
	**β**	**95% CI**	***p*-Value**	**OR**	**95% CI**	***p*-Value**
**Baseline**
PHQ-9 score	−0.018	−0.084,0.048	0.594	1.009	0.970, 1.049	0.650
Depression status
No (ref)
Yes	−0.338	−0.949, 0.274	0.279	1.084	0.764, 1.540	0.651
**Follow up**
PHQ-9 score increase (*i*)	0.157	0.092, 0.221	<0.001^*^	1.063	1.025, 1.103	0.001^*^
Worsening
None (ref)						
Yes	0.650	0.039, 1.261	0.037^*^	1.573	1.113, 2.223	0.010 ^*^

* *p < 0.05*.

*The model adjusted city (Chuzhou, Lu'an and Xuancheng), age ( ≤ 70 and >70), sex (male and female), education (0, 1–6 years and more than 6 years), marital status (married and others), annual income(Lower than 6,500 RMR and 6,500 RMR or higher), BMI (continuous), drinking status (never, former and current), current smoker (Yes and No), Physical activity (daily sitting time), self-rated sleeping quality (very good, good and not good), diabetes (yes and no), hypertension (yes and no), living alone (yes and no), PHQ-9 score at baseline*.

a *Multivariable linear regression analysis included 1,477 participants*.

b *Multivariable logistic regression analysis included 983 participants*.

*j = M – E (M = MMSE score at baseline; E = MMSE score at follow-up)*.

*i = P – Q (P = PHQ-9 score at follow up; Q = PHQ-9 score at baseline)*.

Table 3 shows the results of the sex-stratified analysis. No significant association was detected between baseline depression and cognitive decline and 2-year MCI incidence for either males or females. For males, PHQ-9 score increases were associated with cognitive decline (β = 0.277, 95% CI: 0.128, 0.326, *p* < 0.001) and 2-year MCI incidence (OR = 1.103, 95% CI: 1.041, 1.168, *p* = 0.001), whereas PHQ-9 score increases were associated with only 2-year cognitive decline among females (β = 0.103, 95% CI: 0.015, 0.190, *p* = 0.022). Experienced worsening depression symptoms was associated with a significant larger decline in the 2-year MMSE score in males (β = 1.016, 95% CI: 0.135, 1.897, *p* = 0.024) but not in females.

**Table 3:** The associations between depression and cognitive function by sexes: Coefficients and 95% CIs for 2-year cognitive decline and Odds Ratios and 95% CIs for 2-year MCI incidence.

	**2-year cognitive decline (** ***j*** **)** ^a^			**2-year MCI incidence** ^b^		
	**β**	**95% CI**	***p*-Value**	**OR**	**95% CI**	***p*-Value**
**Male**
**Baseline**
PHQ-9 score	0.037	−0.067, 0.142	0.483	1.022	0.963, 1.084	0.475
Depression status
No (ref)						
Yes	0.008	−0.898, 0.915	0.986	1.259	0.743, 2.132	0.392
**Follow up**
PHQ-9 score increase (*i*)	0.227	0.128, 0.326	<0.001^*^	1.103	1.041, 1.168	0.001^*^
Worsening
None (ref)						
Yes	1.016	0.135, 1.897	0.024^*^	1.610	0.973, 2.666	0.064
**Female**
**Baseline**
PHQ-9 score	−0.060	−0.147, 0.028	0.181	1.009	0.956,1.065	0.736
Depression status
No (ref)						
Yes	−0.657	−1.499, 0.185	0.126	1.043	0.643, 1.693	0.865
**Follow up**
PHQ-9 score increase (*i*)	0.103	0.015, 0.190	0.022^*^	1.039	0.990, 1.093	0.136
Worsening
None (ref)						
Yes	0.290	−0.574, 1.154	0.510	1.615	0.986, 2.647	0.057

* *p < 0.05*.

*The model adjusted city (Chuzhou, Lu'an and Xuancheng), age ( ≤ 70 and >70), sex (male and female), education (0, 1–6 years and more than 6 years), marital status (married and others), annual income (Lower than 6,500 RMR and 6,500 RMR or higher), BMI (continuous), drinking status (never, former and current), current smoker (Yes and No), Physical activity (daily sitting time), self-rated sleeping quality (very good, good and not good), diabetes (yes and no), hypertension (yes and no), living alone (yes and no), PHQ-9 score at baseline*.

a *Multivariable linear regression analysis included 1,477 participants*.

b *Multivariable logistic regression analysis included 983 participants*.

*j = M – E (M = MMSE score at baseline; E = MMSE score at follow-up)*.

*i = P – Q (P = PHQ-9 score at follow up; Q = PHQ-9 score at baseline)*.

The results of the age-stratified analysis are shown in Table 4. For both the older group (aged 70 and older) and younger group (aged under 70), baseline depression status was associated with neither 2-year cognitive decline nor MCI incidence. For both of the age-stratified groups, PHQ-9 score increases were associated with 2-year cognitive decline: for the participants who were under 70, a 1-unit PHQ-9 score increase was significantly associated with a 2-year MMSE score decline of 0.135 (β = 0.135, 95% CI: 0.051,0.220, *p* = 0.002), and for those who were aged 70 and older, a 1-unit PHQ-9 score increase was significantly associated with a 0.168-unit MMSE score decline (β = 0.168, 95% CI: 0.066, 0.270, *p* = 0.001). Whereas PHQ-9 score increases were only associated with 2-year MCI incidence in older group (OR = 1.062, 95% CI: 1.005, 1.222, *p* = 0.033) but not in younger group. Worsening depressive symptoms was not statistically associated with 2-year cognitive decline or MCI incidence for either the older or younger group.

**Table 4:** The associations between depression and cognitive function by age: Coefficients and 95% CIs for 2-year cognitive decline and Odds Ratios and 95% CIs for 2-year MCI incidence.

	**2-year cognitive decline (*****j***)^a^			**2-year MCI incidence** ^b^		
	**β**	**95% CI**	***p*-Value**	**OR**	**95% CI**	***p*-Value**
**Younger (aged under 70)**
**Baseline**
PHQ-9 score	−0.038	−0.126, 0.049	0.390	1.033	0.974, 1.095	0.282
Depression status
No (ref)						
Yes	−0.236	−1.015, 0.543	0.553	1.209	0.743, 1.968	0.445
**Follow up**
PHQ-9 score increase (*i*)	0.135	0.051, 0.220	0.002^*^	1.051	0.999, 1.108	0.056
Worsening
None (ref)						
Yes	0.439	−0.318, 1.196	0.255	1.498	0.927, 2.421	0.099
**Older (aged 70 or older)**
**Baseline**
PHQ-9 score	0.016	−0.087, 0.118	0.765	0.996	0.944, 1.051	0.880
Depression status
No (ref)						
Yes	−0.306	−1.286, 0.673	0.539	1.005	0.595, 1.699	0.985
**Follow up**
PHQ-9 score increase (*i*)	0.168	0.066, 0.270	0.001^*^	1.062	1.005, 1.122	0.033^*^
Worsening
None (ref)						
Yes	0.838	−0.183, 1.858	0.107	1.513	0.896, 2.554	0.121

* *p < 0.05*.

*The model adjusted city (Chuzhou, Lu'an and Xuancheng), age ( ≤ 70 and >70), sex (male and female), education (0, 1–6 years and more than 6 years), marital status (married and others), annual income (Lower than 6,500 RMR and 6,500 RMR or higher), BMI (continuous), drinking status (never, former and current), current smoker (Yes and No), Physical activity (daily sitting time), self-rated sleeping quality (very good, good and not good), diabetes (yes and no), hypertension (yes and no), living alone (yes and no), PHQ-9 score at baseline*.

a *Multivariable linear regression analysis included 1,477 participants*.

b *Multivariable logistic regression analysis included 983 participants*.

*j = M – E (M = MMSE score at baseline; E = MMSE score at follow-up)*.

*i = P – Q (P = PHQ-9 score at follow up; Q = PHQ-9 score at baseline)*.

The results of the analysis stratified by different education levels are shown in Supplementary Table S1. Baseline depression was not associated with cognitive decline and 2-year MCI incidence in the three subgroups. For those who were categorized into the low education group, PHQ-9 score increases were significantly associated with cognitive decline (β = 0.124, 95% CI: 0.036, 0.212, *p* = 0.006) and 2-year MCI incidence (OR = 1.066, 95% CI: 1.018, 1.118, *p* = 0.007), whereas this factor was only associated with 2-year cognitive decline (β = 0.207, 95% CI: 0.097, 0.317, *p* < 0.001) among the participants who had a medium education level. For those who had a high education level, PHQ-9 score increases were only associated with 2-year MCI incidence (OR = 1.272, 95% CI: 1.028, 1.573, *p* = 0.027). Experiencing worsening depressive symptoms was associated with elevated risks of 2-year MCI incident only among those who were categorized in the low education group (OR = 2.184, 95% CI: 1.362, 3.503, *p* = 0.001).

## Discussion

In this longitudinal study with 2-year follow-up, aggravated depressive symptoms were associated with greater cognitive decline and an increased MCI incidence among rural elderly people. Many previous studies have suggested a higher prevalence of depression and cognitive impairment in rural areas than in urban areas in China (38–42). Considering the relatively lower educational level of rural dwellers as well as the barriers due to the constraints of the economy and medical resources in rural regions (43, 44), urban-rural disparities will further expand if effective intervention are not adopted. The current study was the first to investigate the longitudinal associations between depressive symptoms and cognition among Chinese rural elderly individuals.

Consistent with our findings, longitudinal associations between increased depressive symptoms and cognitive decline as well as higher MCI risks have been revealed in many studies (20, 45, 46). However, baseline depression status was not associated with cognitive decline or MCI incidence in this study, which contradicts some studies (21, 47). This might be attributed to the relatively short follow-up duration (2 years) and may also be related to different screening tools for depression assessment, differences in study design and sample size, and heterogeneity of the population.

Similar to many studies (34, 48), in the current study, sex disparities were also found in the associations between depression and cognition, which might be explained through biological or psychosocial pathways. A previous study reported that a type of vascular depression among males might be linked to cerebral vascular pathology, which in turn accelerated the process of cognitive decline, although this phenomenon was not investigated among females (49). Underreported depression symptoms due to the gender bias in reporting depressive symptoms might also contribute to sex disparities since males tend to be unwilling to express their discomfort due to the influences of societal expectations (50). Although some studies reported age-modified associations between depression and cognition (35, 51), in the current study, increased depression symptoms were similarly associated with more cognitive decline among different age groups. Some previous studies reported education-modified associations between depression and cognition (36, 52). Similarly, education level moderated associations between depression symptoms and cognition decline were also detected in the current study.

The strengths of the present study include the prospective study design with a 2-year follow-up period and the relatively high follow-up rate. Moreover, depression symptoms were measured at baseline and at follow-up so that the 2-year change in depressive symptoms could be investigated. However, this study also has several limitations. First, the widely used MMSE was used for assessing the cognitive performance of the participants. However, the MMSE is not a diagnostic tool, although the validity of MMSE has been verified by many studies worldwide. This limitation makes it difficult to grasp the true associations between depression symptoms and cognitive decline. A standard diagnostic procedure should be considered in future studies to achieve a more reliable conclusion. Second, reverse causation cannot be totally avoided due to the short period of follow-up. Future studies addressing the long-term relationship between depression and cognition are still needed. Finally, many studies indicated that some neurological and endocrine conditions might affect the associations, for example, stroke (53, 54) and hypothyroidism (55, 56) might lead to both depression and cognitive impairment; however, such conditions were not considered in the present study. Additionally, some genetic factors were not included in the current study. Future studies conducted under a more comprehensive framework with additional measurements such as blood tests and neuroimaging are needed to verify the associations.

## Conclusion

Worsening depressive symptoms were related to more cognitive decline and a higher risk of incident MCI among rural-dwelling Chinese elderly individuals. Our study highlighted the great importance of depression intervention for preventing cognitive impairment in Chinese elderly individuals living in rural areas. Considering the significant vulnerability of Chinese rural-dwelling elderly individuals, special attention should be given to providing health services regarding effective interventions to reduce depression to combat AD challenges in the future.

## Data Availability Statement

The original contributions presented in the study are included in the article/Supplementary Material, further inquiries can be directed to the corresponding authors.

## Author Contributions

SZ drafted the manuscript. SH, GS, and YZ framed the concept and designed the study. The data collection and material preparation were conducted by SZ, QioW, QinW, and JZ and the data analysis was performed by FH and YZ. All authors meet the criteria for authorship according to their contributions to the manuscript. All authors contributed to the article and approved the submitted version.

## Funding

This research was funded by the National Natural Science Foundation of China, Grant Number 72004003 to YZ, the Key Projects of Science and the Key Project of Science and Technology of Anhui Province, Grant Number 2019b11030012 to SH, Gant Number 202004b11020019 to GS, and the Hefei Municipal Natural Science Foundation, Grant Number 2021005 to GS, and the APC was funded by GS.

## Conflict of Interest

The authors declare that the research was conducted in the absence of any commercial or financial relationships that could be construed as a potential conflict of interest.

## Publisher's Note

All claims expressed in this article are solely those of the authors and do not necessarily represent those of their affiliated organizations, or those of the publisher, the editors and the reviewers. Any product that may be evaluated in this article, or claim that may be made by its manufacturer, is not guaranteed or endorsed by the publisher.
