# Supplementary material for: Depressive Symptoms and Cognitive Decline Among Chinese Rural Elderly Individuals: A Longitudinal Study With 2-Year Follow-Up
Source: Front Public Health. 2022 Jul 13;10:939150. doi: 10.3389/fpubh.2022.939150 (PMC9326072; doi:10.3389/fpubh.2022.939150)
Supplement: Supplementary file 1 [file Table_1.DOCX]

Supplementary Material

**Table S1.** **The associations between depression and cognitive function by education: Coefficients and 95% CIs for 2-year cognitive decline and Odds Ratios and 95% CIs for 2-year MCI incidence**

|  | **2-year cognitive decline ( *j* ) ^a^** | | | **2-year MCI incidence ^b^** | | |
| --- | --- | --- | --- | --- | --- | --- |
|  | **β** | **95% CI** | **P** | **OR** | **95% CI** | **P** |
| **Education**  **(Low: 0 year)** |  |  |  |  |  |  |
| **Baseline** |  |  |  |  |  |  |
| PHQ-9 score | -0.010 | -0.098,0.078 | 0.828 | 1.024 | 0.976,1.075 | 0.336 |
| Depression status |  |  |  |  |  |  |
| No (ref) |  |  |  |  |  |  |
| Yes | -0.339 | -1.174,0.497 | 0.427 | 1.232 | 0.780,1.948 | 0.371 |
| **Follow up** |  |  |  |  |  |  |
| PHQ-9 score increase ( *i* ) | 0.124 | 0.036,0.212 | 0.006* | 1.066 | 1.018,1.118 | 0.007* |
| Worsening |  |  |  |  |  |  |
| None (ref) |  |  |  |  |  |  |
| Yes | 0.615 | -0.244,1.475 | 0.160 | 2.184 | 1.362,3.503 | 0.001* |
| **Education**  **(Medium: 1-6 years)** |  |  |  |  |  |  |
| **Baseline** |  |  |  |  |  |  |
| PHQ-9 score | -0.018 | -0.135,0.100 | 0.760 | 0.981 | 0.903,1.066 | 0.653 |
| Depression status |  |  |  |  |  |  |
| No (ref) |  |  |  |  |  |  |
| Yes | -0.097 | -1.113,0.920 | 0.852 | 0.917 | 0.483,1.740 | 0.792 |
| **Follow up** |  |  |  |  |  |  |
| PHQ-9 score increase ( *i* ) | 0.207 | 0.097,0.317 | 0.000* | 1.012 | 0.938,1.092 | 0.758 |
| Worsening |  |  |  |  |  |  |
| None (ref) |  |  |  |  |  |  |
| Yes  **Education**  **(High: more than 6 years)** | 0.142 | -0.550,1.435 | 0.381 | 0.688 | 0.366,1.294 | 0.246 |
| **Baseline** |  |  |  |  |  |  |
| PHQ-9 score | 0.045 | -0.183,0.273 | 0.698 | 0.931 | 0.744,1.164 | 0.531 |
| Depression status |  |  |  |  |  |  |
| No (ref) |  |  |  |  |  |  |
| Yes | -0.522 | -2.575,1.531 | 0.616 | 0.801 | 0.121,5.289 | 0.818 |
| **Follow up** |  |  |  |  |  |  |
| PHQ-9 score increase ( *i* ) | 0.115 | -0.110,0.339 | 0.314 | 1.272 | 1.028,1.573 | 0.027* |
| Worsening |  |  |  |  |  |  |
| None (ref) |  |  |  |  |  |  |
| Yes | 0.565 | -1.327,2.457 | 0.556 | 3.293 | 0.784,13.836 | 0.104 |

* *p* < 0.05

The model adjusted city (Chuzhou, Lu’an and Xuancheng), age (≤70 and >70), sex (male and female), education (0, 1-6 years and more than 6 years), marital status (married and others), annual income (Lower than 6500 RMR and 6500 RMR or higher), BMI (continuous), drinking status (never, former and current), current smoker (Yes and No), Physical activity (daily sitting time), self-rated sleeping quality (very good, good and not good) ,diabetes (yes and no), hypertension (yes and no), living alone (yes and no), PHQ-9 score at baseline.

^a^ Multivariable linear regression analysis included 1477 participants.

^b^ Multivariable logistic regression analysis included 983 participants.

*j* = *M - E (M* = MMSE score at baseline; *E* = MMSE score at follow-up)

*i = P - Q (P* = PHQ-9 score at follow up; *Q* = PHQ-9 score at baseline)
